# Cognitive Flexibility in Bilinguals and Multilinguals and Its Implications for Dementia

**DOI:** 10.1002/alz.089728

**Published:** 2025-01-03

**Authors:** Preetie Shetty Akkunje, Keerthana Ramesh, Sasi Kumar Archa, Prasanna Suresh Hegde, Michael H. Thaut

**Affiliations:** ^1^ Department of Audiology and Speech Language Pathology, Kasturba Medical College Mangalore, Manipal Academy of Higher Education, Manipal, Karnataka India; ^2^ Dr. Moopens Medical College, Wayanad, Kerala India; ^3^ Naseema Institute of Speech and Hearing, Bangalore, Karnataka India; ^4^ Sukino Inpatient Rehabilitation Centre, Bangalore, Karnataka India; ^5^ Department of Deglutology and Speech Language Pathology, HCG Hospital, Bangalore, Karnataka India; ^6^ Music and Health Science Research Collaboratory, University of Toronto, ON Canada

## Abstract

**Background:**

Verbal fluency (VF) is crucial for language processing and cognitive flexibility, involving selective attention, inhibition, set shifting, response generation, and self‐monitoring. VF assessment includes two distinct tasks, i.e., phonemic and semantic VF. It assesses long‐term verbal semantic memory, phonological awareness, lexical–semantic access, and executive function. Semantic VF is mainly based on generating semantic associations, whereas phonemic VF is based majorly on executive functions instead of lexicon–semantic networks. Even though the VF involves several neural substrates majorly is coordinated by superior frontal lobe and temporal lobe for phonemic and semantic VF, respectively. This study aims to assess VF in linguistically diverse young adults of bilingual and multilingual individuals and its contributing insights into language‐cognition interplay.

**Method:**

A prospective cross‐sectional research study recruited 120 participants, divided into two groups: bilinguals (proficient in at least two languages;n = 60) and multilinguals (proficient in at least three languages;n = 60), identified through the Language Experience and Proficiency Questionnaire. Inclusion criteria encompassed participants aged 20‐50, of both genders, with standard education up to graduation. Participants with neurological or psychiatric language disorders and those scoring ≤25 on the Montreal Cognitive Assessment were excluded from the study. Research‐specific assessments, including Spontaneous speech of Western Aphasia Battery, Addenbrooke’s Cognitive Examination‐III for cognitive functions and VF tests (phonemic & semantic VF, phonemic & semantic switching tasks, alternating language task, controlled semantic association task), were conducted in L1, L2 for bilinguals and L1, L2, and L3 for multilinguals. Statistical evaluation was done using SPSS‐version‐29.0.10, utilizing descriptive statistics, Likelihood ratio, Chi‐square, and Independent sample t’‐tests.

**Results:**

Participants recruited showed no significant difference between groups for age, gender and education highlighting linguistic‐diversity of participants which is essential for comprehending potential variations in research findings and their general applicability. The results revealed that bilinguals outperformed multilinguals in semantic VF tasks while multilinguals outperformed bilinguals in phonemic verbal VF tasks.

**Conclusion:**

The adaptability in switching between semantic fields and linguistic‐dimensions of temporal lobe, particularly evident in bilinguals, underscores the cognitive advantages of bilingualism. These findings are crucial for formulating targeted interventions tailored to the unique needs of linguistically‐diverse dementia patients, to strengthen neural‐networks which can account for the principles of neuroplasticity.

